# The anti-inflammatory and antioxidant effects of melatonin on LPS-stimulated bovine mammary epithelial cells

**DOI:** 10.1371/journal.pone.0178525

**Published:** 2017-05-25

**Authors:** Guang-Min Yu, Hirokazu Kubota, Miki Okita, Teruo Maeda

**Affiliations:** 1 Department of Bioresource Science, Graduate School of Biosphere Science, Hiroshima University, Higashi-Hiroshima, Japan; 2 Setouchi Field Science Center, Graduate School of Biosphere Science, Hiroshima University, Higashi-Hiroshima, Japan; 3 The Research Center for Animal Science, Hiroshima University, Higashi-Hiroshima, Japan; Universidade de Sao Paulo, BRAZIL

## Abstract

Mastitis is the most prevalent disease in dairy cattle worldwide and not only causes huge economic losses in the dairy industry but also threatens public health. To evaluate the therapeutic potential of melatonin in mastitis, we examined the ability of melatonin to protect bovine mammary epithelial cells (bMECs) from the harmful effects of lipopolysaccharide (LPS). We found that melatonin inhibited the LPS-binding protein–CD14–TLR4 signaling pathway in bMECs, which had opposing effects on pro-inflammatory and anti-inflammatory mediators. Melatonin decreased LPS-induced expression of pro-inflammatory cytokines, chemokines, and positive acute-phase proteins (APPs), including tumor necrosis factor-α, interleukin (IL)-1*β*, IL-6, granulocyte-monocyte colony-stimulating factor, chemokine CC motif ligand (CCL)2, CCL5, serum amyloid A, haptoglobin, C-reactive protein, ceruloplasmin, and α-1 antitrypsin, and increased expression of the anti-inflammatory cytokine IL-1Ra and the negative APP fibrinogen. In addition, melatonin increased dityrosine levels but suppressed nitrite levels by upregulating the expression of Nrf2 and heme oxygenase-1 in the Nrf2 antioxidant defense pathway. Finally, melatonin administration increased the viability of LPS-stimulated bMECs. These results suggest that melatonin protects bMECs from LPS-induced inflammatory and oxidant stress damage and provide evidence that melatonin might have therapeutic utility in mastitis.

## Introduction

Despite worldwide efforts to reduce its prevalence, mastitis remains a major economic threat to the dairy industry [1]. It has been reported that the economic losses associated with clinical mastitis range from €61 to €97 per farmed cow per year, due to a combination of the reduction of milk output and the cost of controlling the disease [2]. Mastitis is a complex disease. Its incidence is influenced by microorganisms, the surrounding environment, and the defense mechanism in the udder tissue [3]. Two distinct patterns in the epidemiology of mastitis have been reported. The first is a contagious disease pattern in which pathogenic microorganisms are transferred from animal to animal, and the second is a pattern of opportunistic microorganisms [4]. Inflammatory reactions accompanying mastitis cause severe damage to the mammary secretory epithelium, and as a consequence, reduce milk yield [5].

Gram-negative bacteria are the most frequent cause of mastitis [6]. Lipopolysaccharide (LPS) released from gram-negative bacteria is considered to be an important stimulus of bovine mammary cell (bMEC) inflammation [7]. LPS induces intracellular signaling via the cell surface receptor Toll-like receptor 4 (TLR4) in collaboration with other molecules, such as LPS-binding protein (LBP) and cluster of differentiation 14 (CD14) [8]. TLR engagement activates myeloid differentiation factor 88 (MyD88)-dependent and -independent intracellular signaling pathways [9]. Both signaling pathways induce activation of nuclear factor-κB (NF-κB), resulting in the release of inflammatory cytokines and chemokines [10]. It has been reported that cytokines, such as tumor necrosis factor-α (TNF-α), interleukin (IL)-1*β*, IL-6, IL-8, and granulocyte-macrophage colony-stimulating factor (GM-CSF) [11], and chemokines, such as chemokine CC motif ligand (CCL)2, CCL3, CCL5, CCL20, chemokine CXC motif ligand (CXCL)1, and CXCL2, play important roles in the pathogenesis of mastitis [12]. The levels of these cytokines and chemokines are increased in gram-negative bacteria-infected and LPS-infused mammary glands [13]. Elevated concentrations of chemokines induce the migration of inflammatory leukocytes into the infected area [14], and cytokines amplify the inflammatory response by inducing the production of acute-phase proteins (APPs) such as serum amyloid A (SAA), haptoglobin, C-reactive protein (CRP), ceruloplasmin, α-1 antitrypsin, and fibrinogen [1,15]. Following the inflammatory stimulus, reactive oxygen species and reactive nitrogen species (ROS/RNS) are produced and act in concert to induce cell damage [16].

Melatonin (N-acetyl-5-methoxytryptamine) has been reported to be involved in various physiological processes in both plants and animals, such as, chronobiotic actions [17], female reproduction [18], innate immunity [19,20], abiotic stress resistance [21], anti-cancer [22,23], antiradiation [24] and antioxidant process [25]. In addition, melatonin metabolites, including cyclic-3-hydroxymelatonin, N-acetyl-N-formyl-5-methoxykynuramine, and N-acetyl-5-methoxykynuramine, have the ability to scavenge ROS and RNS [26]. The reduction in free radical-mediated damage by melatonin thus contributes to its anti-inflammatory effects [27]. A number of studies attest to the anti-inflammatory activity of melatonin both *in vivo* and *in vitro*. Melatonin reduces oxidative damage and suppresses IL-6 mRNA expression after venous infusion of LPS and peptidoglycan in rats [28], and also diminishes the levels of TNF-α, IL-1β, and oxidative stress mediators in different regions of rat brains after intracerebroventricular administration of LPS [29]. In pregnant mice, melatonin reduces LPS-induced increases in TNF-α levels in the maternal serum and fetal brain [30]. Melatonin also decreases TNF-α, IL-1β, IL-6 levels and reduces the number of apoptotic neurons after intraventricular *Klebsiella pneumoniae* injection in rats [31]. Finally, expression of TNF-α, IL-1β, IL-6, IL-8, and IL-10 mRNA in LPS-stimulated RAW264.7 cells is inhibited by melatonin treatment [32].

All of these studies demonstrate the ability of melatonin to suppress pro-inflammatory cytokine levels and reduce oxidative stress in experimental inflammation. To our knowledge, however, there have been only two investigations that have addressed the role of melatonin on mastitis model. Boulanger et al. evaluated the effect of melatonin and a proteinase inhibitor in bovine neutrophil-induced mammary cell damage [33]. Recently, a Chinese research team reported the protective effect of melatonin on a mouse mastitis model [34]. Here, we conducted a series of experiments to determine whether melatonin can protect bMECs from LPS-induced cell damage. We investigated the effects of melatonin on the mRNA levels of pro- and anti-inflammatory cytokines, chemokines, and positive and negative APPs in LPS-stimulated bMECs. We also examined ROS/RNS levels in the cell culture medium and evaluated the potential benefits of melatonin on the expression of anti- and pro-apoptotic regulators and cell viability following LPS treatment. Finally, we investigated activation of the TLR4 and Nrf2 signaling pathways to uncover the molecular mechanisms underlying the protective function of melatonin in LPS-induced bMEC inflammation.

## Materials and methods

### Animals

All 12 lactating Holstein cows used in this study were handled in accordance with the regulations of Hiroshima University for animal experiments, and all experimental procedures were approved by the animal care committee of Hiroshima University.

### Cell culture and treatment

The isolation and purification of epithelial cells from milk was performed as described previously [5]. In brief, fresh milk from cows (800 mL/cow) with no clinical signs of mastitis and free of detectable bacteria in the milk was shipped to the laboratory in a thermos flask (28–32°C) within 15 min of collection. The milk sample was centrifuged in 50 mL tubes for 15 min at 1,800 *g*. Cell pellets were resuspended in 12 mL Dulbecco’s phosphate-buffered saline (DPBS; Nissui Pharmaceutical, Tokyo, Japan), and transferred to 15 mL tubes. The cells were washed three times by resuspension in DPBS and centrifugation for 5 min at 500 g. After washing, the cells from each cow were seeded in 5 mL DMEM/F12 medium (Gibco BRL/Invitrogen, Karlsruhe, Germany) containing 10% fetal bovine serum (FBS; Sigma-Aldrich, St. Louis, MO, USA), 1% nucleosides (Millipore, Billerica, MA, USA), 1% non-essential amino acids (Gibco BRL/Invitrogen), 1 mM sodium pyruvate (Gibco BRL/Invitrogen), and 1% antibiotic-antimycotic mixed stock solution (Nacalai Tesque, Kyoto, Japan). Cells were cultured at 37°C in an atmosphere of 90% humidity and 5% CO_2_. The medium was changed every 48 h. When cells reached confluence (~3 weeks), they were digested and propagated using 1 mL dissociation solution (CTK; ReproCELL, Yokohama, Japan). All experiments were performed with cells at the second passage.

A stock solution was prepared by dissolving 5 mg of melatonin (Sigma-Aldrich) in 100 μL ethanol. The working concentrations were prepared by dilution in cell culture medium to give final concentrations of 43 and 430 μM. As controls, cells were incubated with ethanol at the highest concentration used for the melatonin treatment. Cells were pre-incubated with or without melatonin for 12 h and then stimulated for 12 h with 100 ng/mL LPS (ALX-581-014-L001; Enzo Life Sciences, Farmingdale, NY, USA), a concentration determined by our trial test (S1 Fig).

### RNA isolation, reverse transcription and quantitative real-time PCR (qRT-PCR)

Total RNA was extracted using NucleoSpin RNA (Macherey-Nagel, Düren, Germany) according to the manufacturer's protocol. RNA concentrations were calculated from the optical density at 260 nm, assuming an OD260 unit is equivalent to 40 μg/mL RNA. The RNA purity was determined by measuring the absorbance ratio at 260/280 nm. Only samples with a ratio > 1.8 were used. Aliquots of 240 ng of total RNA were reverse transcribed using ReverTra Ace (Toyobo, Osaka, Japan).

qRT-PCR was performed using SYBR Premix Ex Taq II (Takara Bio) on an Applied Biosystems StepOne Real-Time PCR System (Life Technologies) according to the method described previously [35]. In brief, the qPCR mixture consisted of 10 μL SYBR Premix Ex Taq II, 0.4 μM forward and reverse primers, 0.4 μL ROX Reference Dye, 2 μL template, and ddH_2_O to a total volume of 20 μL. The amplification parameters were as follows: initial denaturation at 95°C for 30 s followed by 50 cycles of denaturation at 95°C for 5 s, annealing and extension at 60–68°C for 34 s, and a melting curve from 60 to 95°C, increasing in increments of 0.5°C every 5 s. Normalization was performed using the housekeeping gene glyceraldehyde 3-phosphate dehydrogenase (GAPDH) as a control. Primer sequences are listed in Table 1. Relative mRNA expression was calculated by the 2^–ΔΔct^ method. Samples from 5 cows were measured in duplicate.

**Table 1:** Primers used for qRT-PCR.

Genes	Primer sequence (5’–3’)	Product size (bp)	Tm (°C)
GADPH	Forward: GTCTTCACTACCATGGAGAAGG	199	60
	Reverse: TCATGGATGACCTTGGCCAG		
LBP	Forward: GGTGCGCAAGAGGATACTGA	192	60
	Reverse: AAGAGATTCAGCAGCCACCC		
CD14	Forward: GACCTCCGCTGTCTTTCCAG	188	60
	Reverse: CTCGACGGCAACCATACACT		
TLR4	Forward: TCCCCGACAACATCCCCATA	159	64
	Reverse: GGCCCTGAAATGTGTCGTCT		
NF-κB	Forward: CAGCCTGGTGGGAAAACACT	150	60
	Reverse: CAGGCATCTGTCATTCGTGC		
TNF-α	Forward: CCACGTTGTAGCCGACATC	156	60
	Reverse: CCCTGAAGAGGACCTGTGAG		
IL-1β	Forward: CAGTGCCTACGCACATGTCT	174	60
	Reverse: GCCAGCACCAGGGATTTTTG		
IL-6	Forward: GCTGAATCTTCCAAAAATGGAGG	200	60
	Reverse: GCTTCAGGATCTGGATCAGTG		
IL-8	Forward: ATGACTTCCAAGCTGGCTGTTG	149	60
	Reverse: TTGATAAATTTGGGGTGGAAAG		
GM-CSF	Forward: GACTCCCAGGAACCAACGTG	113	60
	Reverse: TCGTAGTGGGTGGCCATCAT		
IL-1Ra	Forward: CTCGAGGTCACAGGATGGACA	136	60
	Reverse: ACATCCCAGATCCTGAAGGC		
CCL2	Forward: TTAACTCCCAAGTCGCCTGC	158	68
	Reverse: CTGCTTGGGGTCTGCACATA		
CCL3	Forward: TGCACTGACGCTCAAGCC	181	60
	Reverse: CGATTTTGCGAGAAAGCTGCC		
CCL5	Forward: GCCTTGAACCTGAACTTGCG	112	64
	Reverse: TGGAATCTGTGCCTTCCCAG		
CCL20	Forward: CAGCAAGTCAGAAGCAAGCA	169	60
	Reverse: CTTTGGATCTGCACACACAGC		
CXCL1	Forward: CCGCCCCCATGGTTAAGAAA	161	60
	Reverse: AAACACAGTCCAGATGGCCC		
CXCL2	Forward: CCAGCTCTAACTGACCAGGTG	116	60
	Reverse: ATGGCCTTAGGAGGTGGTGA		
SAA	Forward: CCTGGGCTGCTAAAGTGATC	184	60
	Reverse: TACTTGTCAGGCAGGCCAG		
haptoglobin	Forward: GCATGCTGGAAATGGGGTGT	141	60
	Reverse: ACCAAGTACTCCACGTAGCC		
ceruloplasmin	Forward: TGACCTCTTCCCTGGGACAT	147	60
	Reverse: CCAGACTTGATCTCTTCGTTTGG		
CRP	Forward: TTGGTGGCACAGACTGACC	176	66
	Reverse: TGAAGGCTTTGAGTGGTGTCC		
α-1 antitrypsin	Forward: GACTTCCATGTGGACGAGCA	122	68
	Reverse: TTGCCCACGTAGTCCAACAG		
fibrinogen	Forward: AAACCGGACCATGACCATCC	176	60
	Reverse: ACGCTCCACCCCAGTAGTAT		
Nrf2	Forward: CATGGCATCACCAGACCACT	130	60
	Reverse: CGGTGTTTTGGGACCCTTCT		
HO-1	Forward: CAAGCGCTATGTTCAGCGAC	198	60
	Reverse: TTGGTGGCACTGGCGATATT		
iNOS	Forward: CTTGTTCTCGAGGTGCCCAT	174	60
	Reverse: GTCCCGGACTCCAACTTCTG		
Bcl-2	Forward: TGGAGGAGCTCTTCAGGGAC	140	64
	Reverse: GTACTCGGTCATCCACAGGG		
Bax	Forward: GCGCATCGGAGATGAATTGG	153	60
	Reverse: TAGAAAAGGGCGACAACCCG		

GAPDH, glyceraldehyde-3-phosphate dehydrogenase; LBP, lipopolysaccharide binding protein; CD14, cluster of differentiation 14; TLR4, Toll-like receptor 4; NF-κB, nuclear factor κB; TNF-α, tumor necrosis factor-α; IL, interleukin; IL-1Ra, IL-1 receptor antagonist; GM-CSF, granulocyte-macrophage colony-stimulating factor; CCL, chemokine CC motif ligand; CXCL, chemokine CXC motif ligand; SAA, serum amyloid A; CRP, C-reactive protein; Nrf2, nuclear factor E2-related factor; HO-1, heme oxygenase 1; iNOS, inducible nitric oxide synthase; Bcl-2, B-cell lymphoma 2; Bax, Bcl-2-associated X protein.

### Enzyme-linked immunosorbent assay (ELISA)

Cell culture medium was pre-cleared by centrifugation at 3,000 g for 20 min to remove cells and then stored at −20°C until use. Dityrosine levels in duplicate samples of culture medium were measured using a competitive ELISA kit (JaICA, Nikken SEIL, Shizuoka, Japan), according to the manufacturer's instructions. Dityrosine concentrations were calculated by comparison with a standard curve that typically ranged from 0.05 to 12 μM.

### The Griess reaction

Levels of the nitric oxide (NO) metabolite nitrite in samples of cell-free culture medium were measured with a nitrite colorimetric assay kit using the Griess reaction (Dojindo Molecular Technologies, Tokyo, Japan), according to the manufacturer's instructions. Samples were measured spectrophotometrically (Model 680 microplate reader S/N 22002; Bio-Rad Laboratories, Hercules, CA, USA) and nitrite concentrations (μM) were calculated with reference to a standard curve. Measurements were performed in duplicate.

### Fluorescence microscopy

Cell viability was determined using a Fluo Cell Double Staining Kit (MoBiTec, Göttingen, Germany). bMECs were placed on a coverslip and incubated for 15 min at 37°C in the presence of calcein-AM (0.4 μM) and propidium iodide (PI, 0.3 μM). The coverslip was placed on a slide and the cells were examined with a Keyence BZ-9000 fluorescence microscope (Keyence, Osaka, Japan). Digital images were captured at 100× magnification.

### Cell viability analysis

The viability of bMECs was quantified using an MTT assay. Briefly, cells were seeded in 96-well plates at 1 × 10^5^ cells/well and incubated with or without melatonin (43 or 430 μM) for 12 h. Medium or 100 ng/mL LPS was then added and the cells were incubated for a further 12 h. The cells were washed three times with DPBS, and 5 mg/mL MTT (Sigma-Aldrich) in DPBS was added to each well. After 4 h incubation at 37°C, the supernatant was removed and 100 μL dimethyl sulfoxide (Nacalai Tesque) was added to stop the reaction. The plates were shaken for 15 min, and the absorbance at 570 nm was measured using a spectrophotometer (Model 680 microplate reader S/N 22002; Bio-Rad Laboratories). The measurements were performed in sextuplicate.

### Statistical analysis

Continuous variables are expressed as the mean ± standard deviation (SD) of 3–6 independent experiments. Statistical analyses were performed using one-way analysis of variance (ANOVA) followed by Duncan’s multiple-range test with Statview software (Abacus Concepts, Berkeley, CA, USA). *P <* 0.05 was considered statistically significant.

## Results

### Melatonin prevents LPS-induced cell death

**Fig 1:**
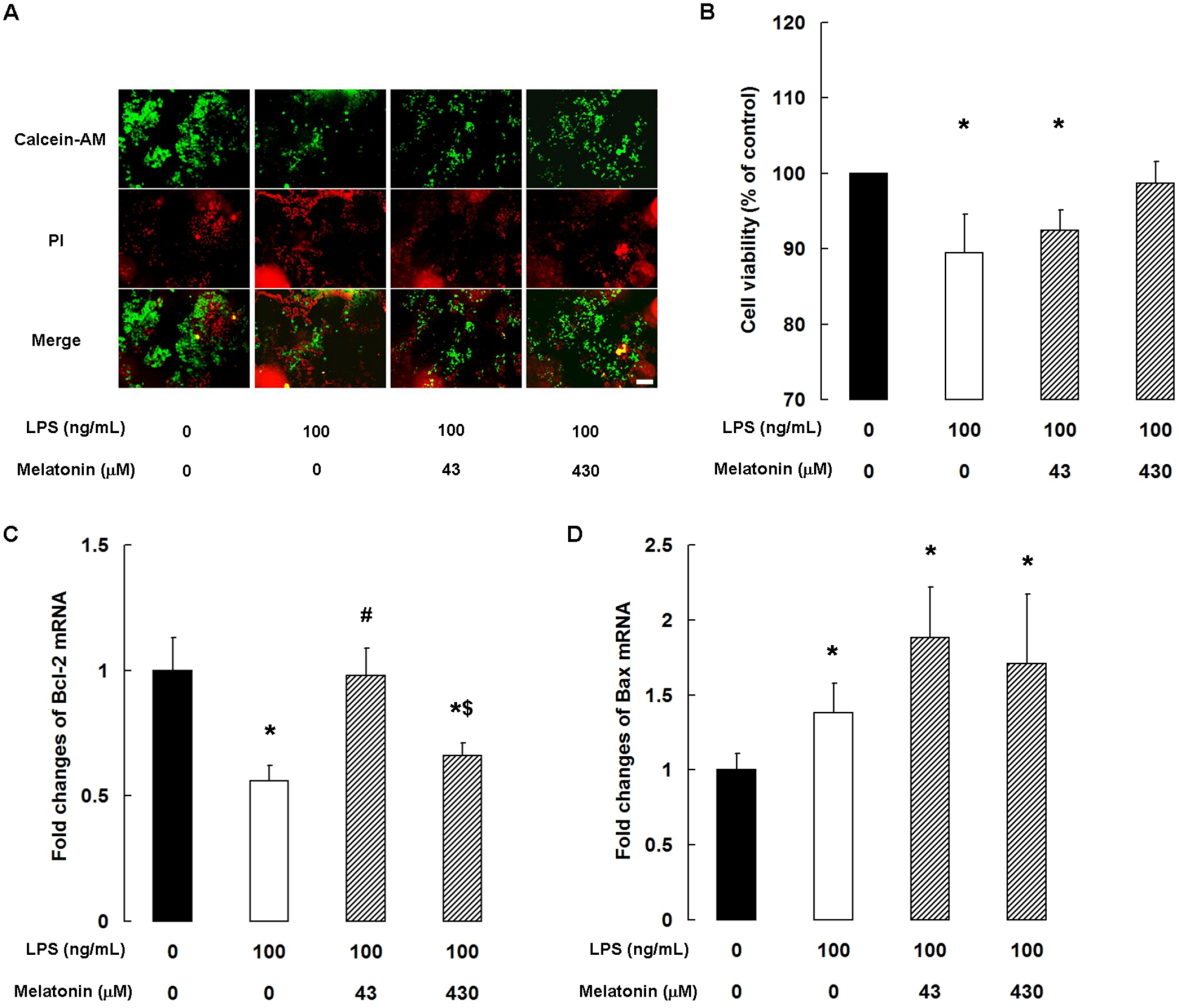
Melatonin prevents LPS-induced death of cultured bMECs. (A) Fluorescence microscopic images of cell double-staining. Data are representative of three independent experiments. Scale bar: 20 μm. (B) cell viability analyzed by MTT assay. Data are the mean ± SD of three independent experiments, each performed in sextuplicate. qPCR analysis of (C) Bcl-2 and (D) Bax. Data are the mean ± SD of five independent experiments, each performed in duplicate. *P < 0.05 vs the control group; ^#^*P* < 0.05 vs the 100 ng/mL LPS group; ^$^*P* < 0.05 vs the 100 ng/mL LPS + 43 μM melatonin group.

We examined the viability of bMECs by co-incubating them with calcein-AM and PI, two fluorescent dyes that label live and dead cells, respectively. We found that although LPS reduced the cell viability, melatonin at concentrations of 43 and 430 μM prevented cell death (Fig 1A). Similarly, quantification of cell viability using the MTT assay revealed that 430 μM melatonin was able to prevent the loss of cell viability caused by LPS stimulation (Fig 1B). The results of qPCR show that LPS stimulation markedly attenuated the expression of Bcl-2 mRNA (*P <* 0.05), but pretreatment with 43 μM melatonin was sufficient to inhibit the effect (Fig 1C). However, while the expression of Bax mRNA was significantly increased by LPS stimulation (*P <* 0.05), there was no significant difference in its expression in cells pre-incubated with or without melatonin (Fig 1D).

### Melatonin inhibits the TLR4 signaling pathway in LPS-stimulated bMECs

**Fig 2:**
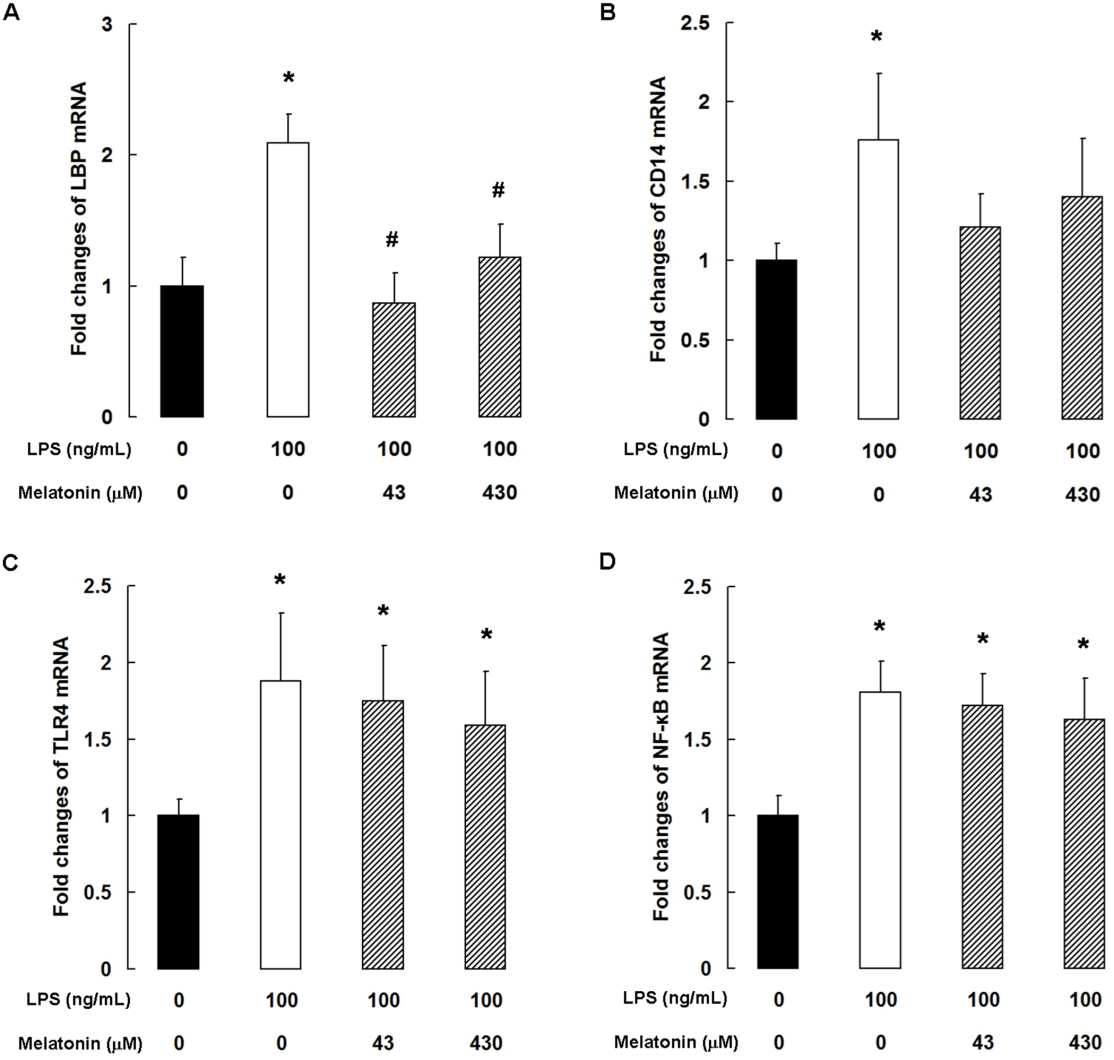
Melatonin inhibits the TLR4 signaling pathway in LPS-stimulated bMECs. qPCR analysis of (A) TLR4, (B) LBP, (C) CD14, and (D) NF-κB. Data are the mean ± SD of five independent experiments, each performed in duplicate. *P < 0.05 vs the control group; ^#^*P* < 0.05 vs the 100 ng/mL LPS group.

As shown in Fig 2, 100 ng/mL LPS significantly increased the expression of LBP, CD14, TLR4, and NF-κB mRNA in bMECs. Melatonin markedly suppressed the expression of LBP mRNA at both concentrations tested (100 ng/mL LPS + 43 or 430 μM melatonin, *P* < 0.05; Fig 2A). CD14 mRNA levels were not significantly different in the control (LPS 0 ng/mL + melatonin 0 μM) group and the 100 ng/mL LPS + 43 or 430 μM melatonin groups (Fig 2B). However, melatonin had no significant effect on the LPS-stimulated increase in TLR4 and NF-κB mRNA (Fig 2C and 2D).

### Melatonin modulates LPS-induced cytokine expression

**Fig 3:**
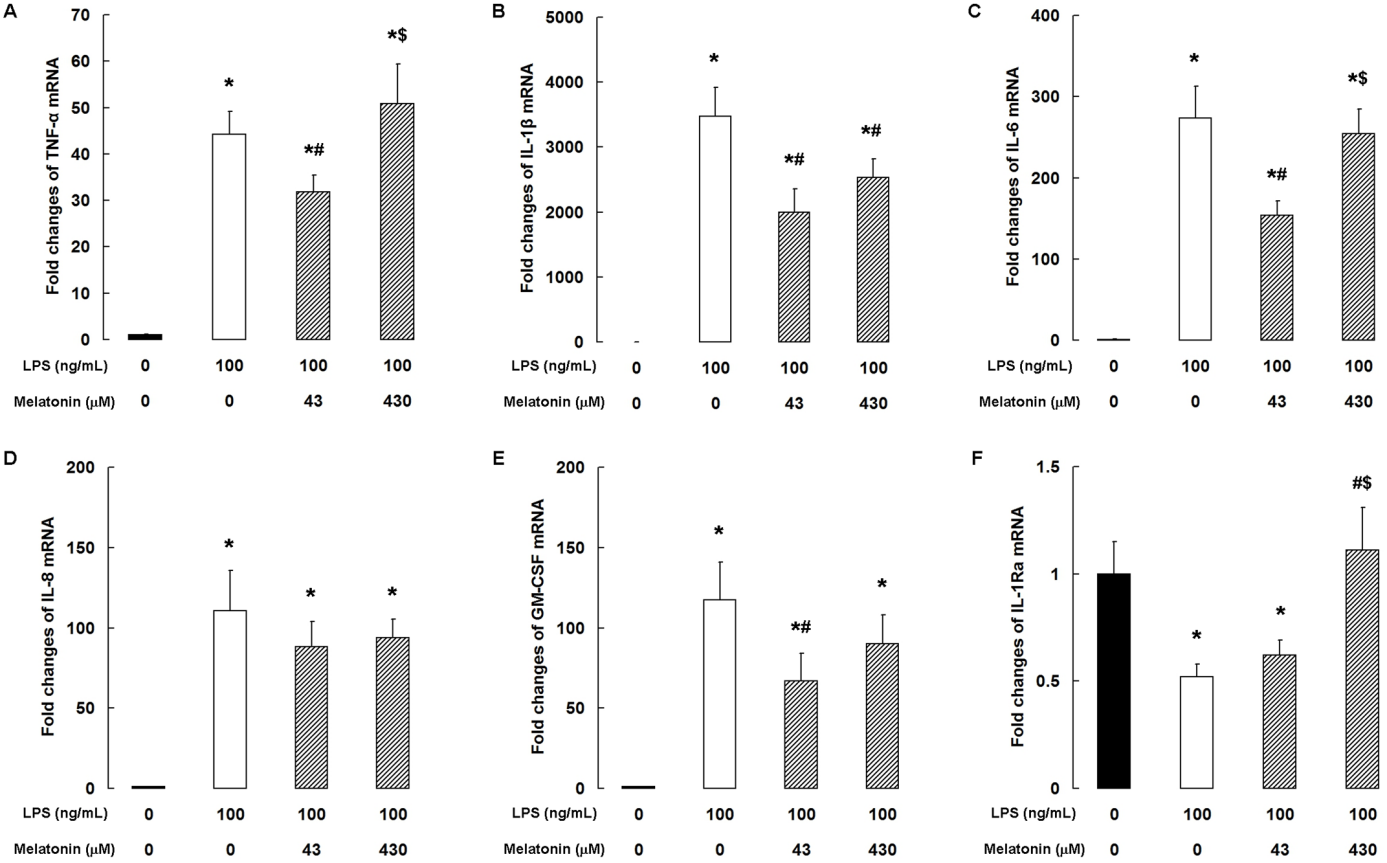
Melatonin modulates LPS-induced cytokine expression. qPCR analysis of (A) TNF-α, (B) IL-1β, (C) IL-6, (D) IL-8, (E) GM-CSF, and (F) IL-1Ra. Data are the mean ± SD of five independent experiments, each performed in duplicate. *P < 0.05 vs the control group; ^#^*P* < 0.05 vs the 100 ng/mL LPS group; ^$^*P* < 0.05 vs the 100 ng/mL LPS + 43 μM melatonin group.

LPS caused substantial increases in the mRNA levels of the pro-inflammatory cytokines TNF-α, IL-1β, IL-6, IL-8, and GM-CSF (Fig 3). Addition of 43 μM melatonin significantly decreased TNF-α, IL-6, and GM-CSF mRNA levels (*P <* 0.05, Fig 3A, 3C and 3E), whereas IL-1β mRNA levels were significantly decreased by melatonin at concentrations of 43 and 430 μM (*P* < 0.05, Fig 3B). However, melatonin had no significant effect on IL-8 mRNA levels in LPS-stimulated cells (Fig 3D). With regard to the anti-inflammatory cytokine IL-1Ra, although LPS treatment markedly decreased IL-1Ra mRNA level, pretreatment with 430 μM melatonin rescued its expression (Fig 3F).

### Melatonin inhibits LPS-induced chemokine expression

**Fig 4:**
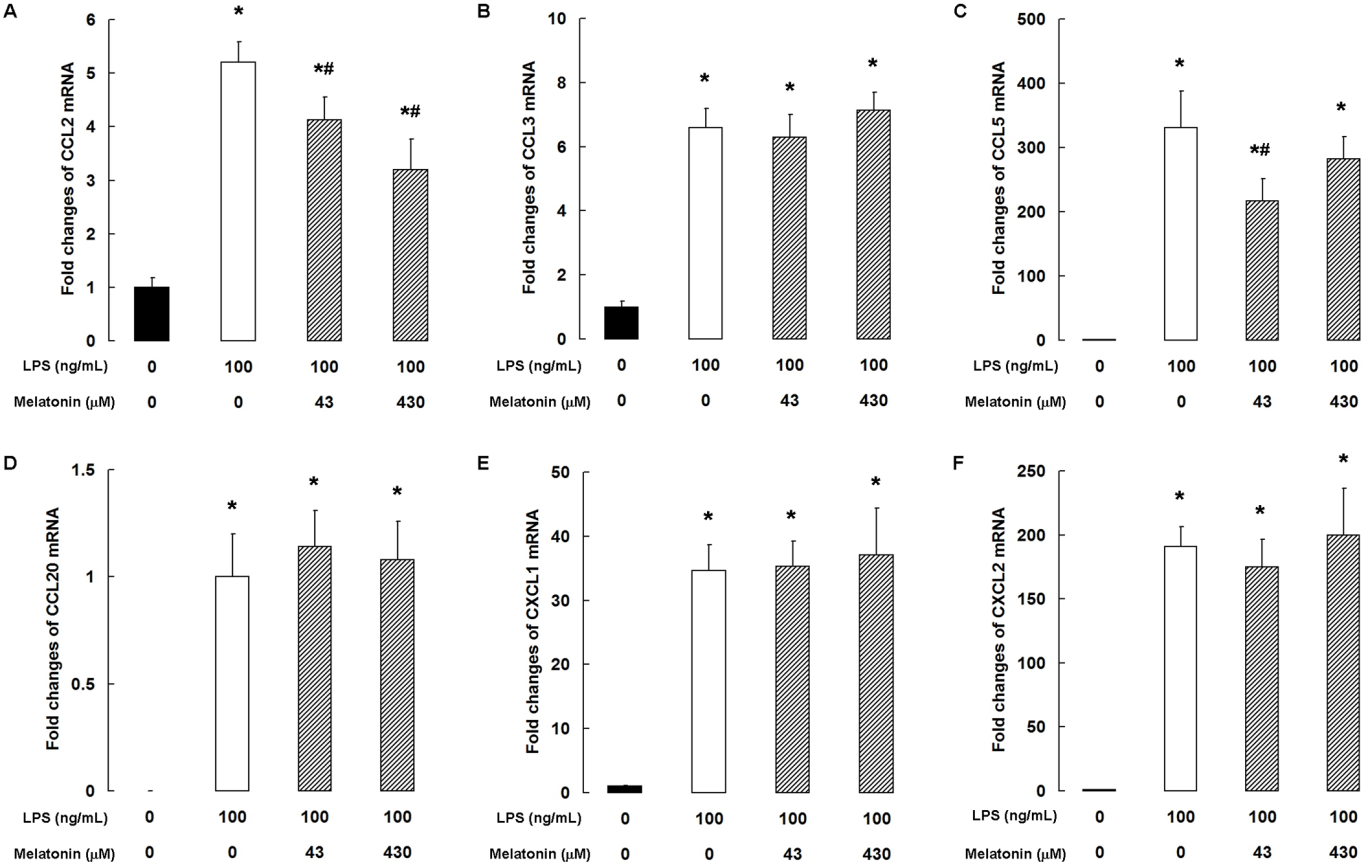
Melatonin inhibits LPS-induced chemokine expression. qPCR analysis of (A) CCL2, (B) CCL3, (C) CCL5, (D) CCL20, (E) CXCL1, and (F) CXCL2. Data are the mean ± SD of five independent experiments, each performed in duplicate. *P < 0.05 vs the control group; ^#^*P* < 0.05 vs the 100 ng/mL LPS group; ^$^*P* < 0.05 vs the 100 ng/mL LPS + 43 μM melatonin group.

LPS stimulation substantially increased the mRNA levels of CCL2, CCL3, CCL5, CCL20, CXCL1, and CXCL2 (Fig 4). Melatonin at both 43 and 430 μM markedly suppressed the expression of CCL2 mRNA (*P <* 0.05, Fig 4A) and 43 μM significantly decreased CCL5 mRNA level (*P <* 0.05, Fig 4C). However, melatonin had no significant effect on the LPS-induced increase in CCL3, CCL20, CXCL1, and CXCL2 mRNA (Fig 4B, 4D, 4E and 4F).

### Melatonin modulates the LPS-induced expression of acute-phase protein

**Fig 5:**
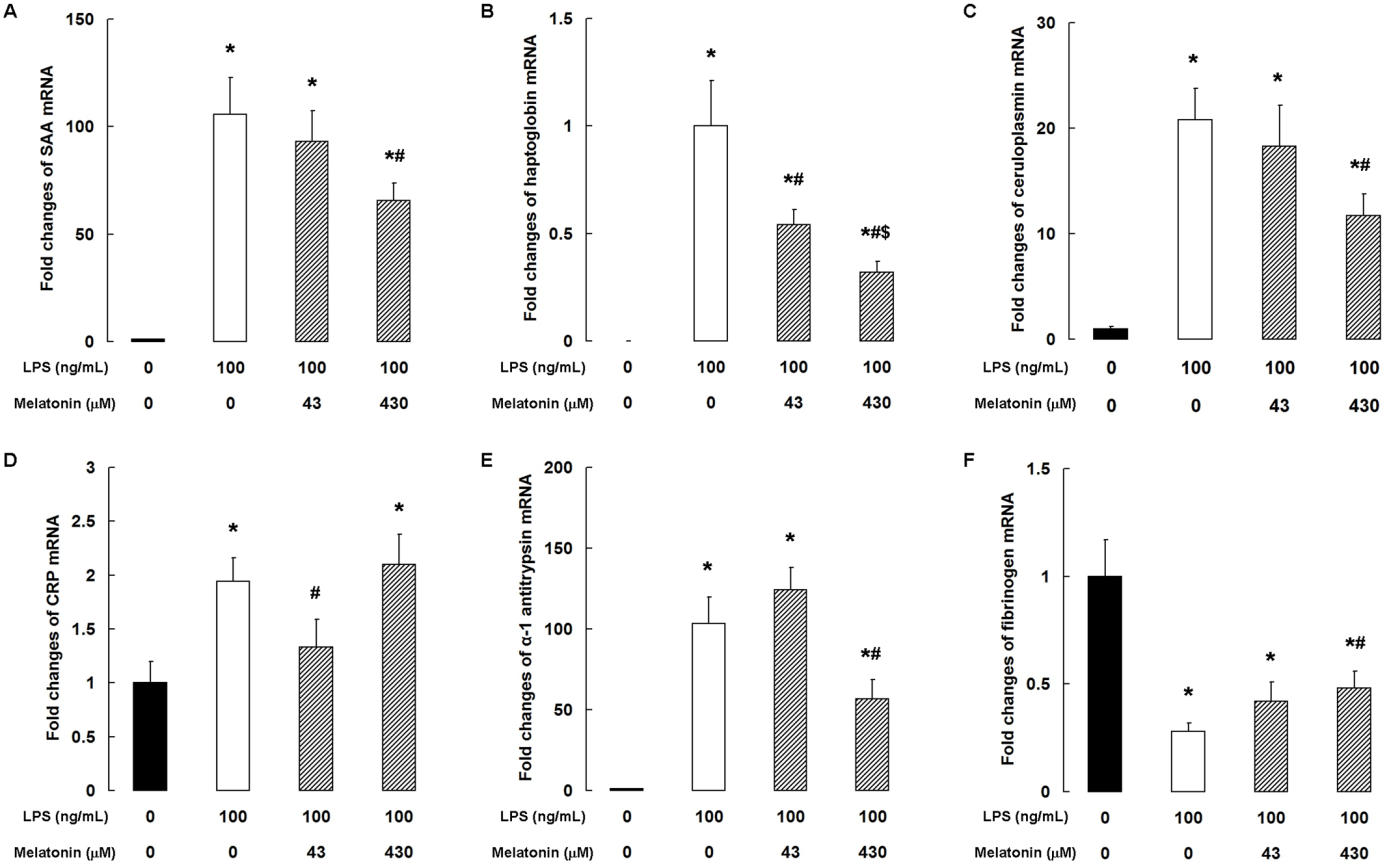
Melatonin modulates LPS-induced acute-phase protein expression. qPCR analysis of (A) SAA, (B) haptoglobin, (C) ceruloplasmin, (D) CRP, (E) α-1 antitrypsin, and (F) fibrinogen. Data are the mean ± SD of five independent experiments, each performed in duplicate. *P < 0.05 vs the control group; ^#^*P* < 0.05 vs the 100 ng/mL LPS group; ^$^*P* < 0.05 vs the 100 ng/mL LPS + 43 μM melatonin group.

LPS treatment clearly increased the expression of SAA, haptoglobin, ceruloplasmin, CRP, and α-1 antitrypsin mRNA (Fig 5). When present at 43 μM, melatonin significantly decreased CRP mRNA level (*P <* 0.05, Fig 5D), and at 430 μM, it markedly attenuated the increase in SAA, ceruloplasmin, and α-1 antitrypsin mRNA levels (*P <* 0.05, Fig 5A, 5C and 5E). In addition, the LPS-induced increase in haptoglobin mRNA was significantly inhibited by melatonin at concentrations of 43 and 430 μM (*P <* 0.05, Fig 5B). In contrast, fibrinogen mRNA level was markedly suppressed by LPS, and this was partially rescued by pretreatment with 430 μM melatonin (*P <* 0.05, Fig 5F).

### Melatonin activates the Nrf2 antioxidant defense pathway in LPS-stimulated bMECs

**Fig 6:**
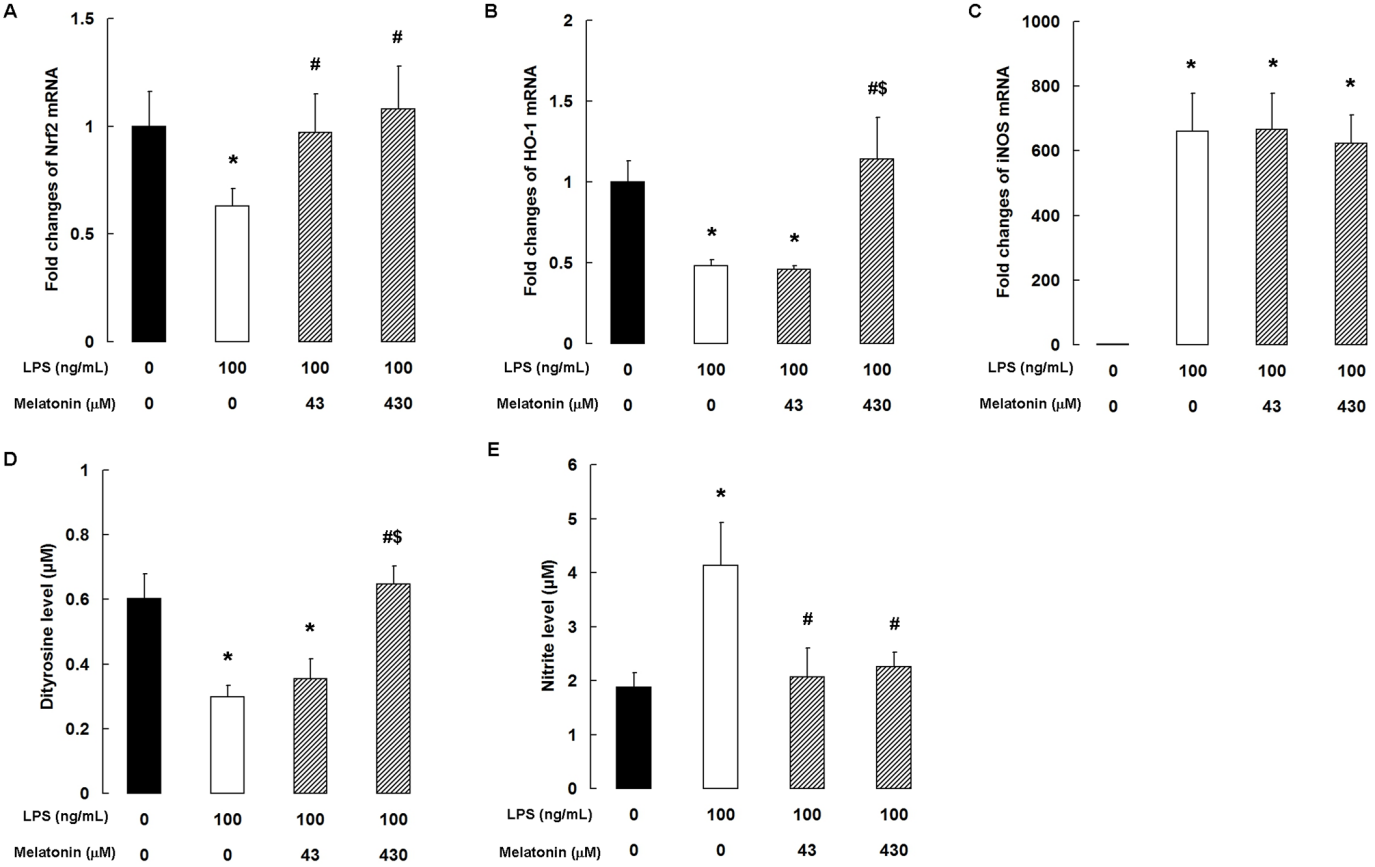
Melatonin reduces oxidative stress in LPS-stimulated bMECs. qPCR analysis of (A) Nrf2, (B) HO-1, and (C) iNOS. Data are the mean ± SD of five independent experiments, each performed in duplicate. (D) dityrosine level and (E) nitrite level in the culture medium. Data are the mean ± SD of six independent experiments, each performed in duplicate. *P < 0.05 vs the control group; ^#^*P* < 0.05 vs the 100 ng/mL LPS group; ^$^*P* < 0.05 vs the 100 ng/mL LPS + 43 μM melatonin group.

As shown in Fig 6, LPS significantly inhibited the expression of Nrf2 and heme oxygenase-1 (HO-1) mRNA in bMECs. Melatonin concentrations of 43 and 430 μM rescued Nrf2 mRNA expression (Fig 6A), while 430 μM melatonin rescued the expression of HO-1 (Fig 6B). Finally, the level of inducible NO synthase (iNOS) mRNA was substantially increased by LPS stimulation; however, melatonin pretreatment had no significant effect (Fig 6C).

### Melatonin inhibits oxidative stress in LPS-stimulated bMECs

To examine oxidative stress, we measured dityrosine and nitrite levels in the supernatants of treated bMECs. ELISA assays showed that LPS substantially decreased the production of dityrosine (*P <* 0.05), but this effect was inhibited by pretreatment with 430 μM melatonin (Fig 6D). The nitrite level in the culture medium, determined by the Griess reaction, was markedly increased by LPS stimulation (Fig 6E, *P <* 0.05). As expected, melatonin significantly suppressed the nitrite levels at both 43 and 430 μM (*P <* 0.05).

## Discussion

Mastitis is the most prevalent disease of dairy cattle worldwide and not only causes huge economic losses in the dairy industry but also threatens public health [2]. The extensive use of antibiotics in dairy herds, however, leads to milk being discarded and increases consumer concern over food safety [36]. At the same time, accompanied with the world population growing, more and better-quality food will be needed [37]. Accumulating evidence attributes the anti-inflammatory effects of melatonin to its direct antioxidant actions. However, the exact mechanism by which melatonin protects against mastitis and the key parameters that it influences remain to be elucidated. The present study confirmed that melatonin inhibits the TLR4 signaling pathway and diminishes mRNA expression of pro-inflammatory cytokines, chemokines, and positive APPs, but increases mRNA expression of an anti-inflammatory cytokine and a negative APP in LPS-stimulated bMECs. In addition, we showed that melatonin inhibits oxidative stress through activation of the Nrf2 antioxidant defense pathway and improves cell viability.

bMECs play an important role in protecting the mammary gland from severe inflammation caused by invading pathogens [5]. After stimulation by molecules released from microorganisms, bMECs produce several immunomodulators that initiate the immune response and activate leukocytes in the udder [38]. In the present study, the bMECs were isolated from fresh milk, a non-invasive method of collection. Cells from individual cows were obtained and cultured separately to provide biological replicates.

Melatonin is involved with the regulation of biological rhythms and control of seasonal reproduction in photoperiodic animals [17]. The application of melatonin to treat inflammation derives from previous researches where melatonin doses drastically exceeding the nocturnal levels (the peak of physiological concentration of melatonin is 0.1 nM in bovine serum and milk [39]) are required to exert clear effects [40]. In fact, melatonin can exert both pro- and anti-inflammatory effects, which seems to largely depend on the cells and systems studied, and especially to the grade of inflammation. The pro-inflammatory effects seem to be observed under basal conditions, whereas the anti-inflammatory effects are observed in the presence of high-grade inflammation [41]. Thus, melatonin appears to act as a buffer, allowing the immune system to respond to infections while attenuating serious damage in high-grade inflammation [42]. It is also thought that melatonin may promote the early stages of inflammation but suppress the sustained response to prevent chronic inflammatory disease [40]. There have been only two investigations that have addressed the role of melatonin on mastitis model before our report. In contrast to the present study, Boulanger et al. used a mammary epithelial cell line to evaluate 1,000 ng/mL LPS-induced oxidative stress response [33]. It is a pretty important work showing the effect of melatonin and catalase on bovine neutrophil-induced mammary epithelial cell damage in a co-culture of bovine neutrophils and MAC-T cells. The anti-inflammatory effect of melatonin is reported on a mouse mastitis model [34]. Melatonin attenuated LPS-induced mammary histopathologic changes and myeloperoxidase activity in that model. Melatonin also inhibited LPS-induced inflammatory cytokines TNF-α, IL-1β and IL-6 production in mouse mammary tissues. *In vitro*, melatonin was found to inhibit 100 ng/mL LPS-induced inflammatory responses by activating peroxisome proliferator-activated receptor-γ in mouse MECs. Prior to the present study, we conducted a trial test which reveals both 100 and 1,000 ng/mL LPS significantly increased the expression of TNF-α and SAA mRNA in bMECs, but there was no significant difference between 100 and 1,000 ng/mL LPS groups (S1 Fig). Thus, we conducted a series of experiments to demonstrate that melatonin protect bMECs from 100 ng/mL LPS-induced inflammatory and oxidative damage. Our findings are consistent with previous observation that modulation of apoptosis requires high melatonin doses [43]. The two investigations mentioned above also show that melatonin at 25–500 μM effectively inhibited the LPS-induced mammary epithelial cell damage.

Cytokine are a broad category of small, nonstructural, secreted proteins that are synthesized by and induce a response in nearly all nucleated cells [44]. They are primarily involved in immune and inflammatory responses, including responses to infection, trauma, and cancer, and also play roles in reproduction. Cytokines can be classified according to their biological activities. Pro-inflammatory cytokines, such as TNF-α, IL-1β, IL-6, IL-8, and GM-CSF studied here, generally exacerbate disease and infections, while anti-inflammatory cytokines, such as IL-1Ra, reduce inflammation and promote healing [45]. Considerable attention has focused on blocking the action of pro-inflammatory cytokines, particularly during overwhelming infection. Previous studies have shown that melatonin suppresses TNF-α, IL-1β, IL-6, IL-8, and IL-10 during bacterial infections or LPS treatment *in vitro* and *in vivo* [28–32,34]. The results of the present study suggest that melatonin decreases expression of TNF-α, IL-1β, IL-6, and GM-CSF mRNA in LPS-stimulated bMECs. To our knowledge, this is the first demonstration of an inhibitory effect of melatonin on GM-CSF expression. We also report for the first time that LPS-mediated suppression of IL-1Ra can be reversed by melatonin, which is likely to have a beneficial effect by reducing inflammatory responses.

Chemokines are chemotactic cytokines that induce leukocyte migration by interacting with G protein-coupled receptors. Chemokines play fundamental roles in the development, homeostasis, and function of the immune system [46]. During infection, microbial antigens initiating inflammatory responses provoke the production of numerous chemokines by local cells expressing TLRs. Effector leukocytes in the blood are attracted by chemokines and travel along the increasing chemokine concentration gradient towards the site of infection [47]. Earlier work has described only the effect of melatonin on CXCL8 (also known as IL-8) and CXCL10 [32,48]. The present study is the first to examine the effect of melatonin on CCL2, CCL3, CCL5, CCL20, CXCL1, and CXCL2 mRNA expression in LPS-stimulated bMECs. We note that only CCL2 and CCL5 were suppressed. An understanding of the effect of melatonin on chemokines should help to define future directions in chemokine-based anti-inflammatory therapies. It is well recognized that the TLR4 signaling pathway is modulated by melatonin under inflammatory conditions [48,49]. In the present study, melatonin reduced the transcription of LBP and CD14, which collaborate with TLR4 on the cell surface to activate intracellular inflammatory signaling pathways.

The acute-phase response is a series of non-specific and complex reactions occurring soon after the onset of stress, injury, trauma, infection, inflammation, and neoplasia, which aim to eliminate the infectious agents, restore homeostasis, and promote healing [1]. It is a highly coordinated process comprising a wide variety of behavioral, physiological, biochemical, and nutritional changes [50]. The most important metabolic change is the significant increase or decrease in production of APPs (positive and negative APPs, respectively) [51]. The marked changes in APP levels that occur during infection and inflammation allows them to serve as important diagnostic and prognostic markers for various infectious and inflammatory diseases.^1^ In the present study, we investigated the effect of melatonin on expression of the positive APPs SAA, haptoglobin, CRP, ceruloplasmin, and α-1 antitrypsin, and of the negative APP fibrinogen in LPS-stimulated bMECs. Only the effect of melatonin on CRP has been described previously, in a study of diabetes-associated low-grade inflammation [52]. We found that melatonin reduced the expression of all of the positive APPs and significantly increased the mRNA level of the negative APP.

An inflammatory response activates the synthesis of immunomodulators that result in cataclysmic levels of ROS and RNS [16]. These toxicants trigger the discharge of previously sequestered Ca^2+^ into the cytosol and cause mitochondrial lesions that result in the release of cytochrome c and activation of the apoptotic cascade [25]. An effective anti-inflammatory therapeutic strategy needs to be able to reduce the production of inflammatory mediators and to abate oxidative stress initiated by inflammation [40]. Melatonin is a powerful antioxidant. It represses oxidative stress by direct scavenging of free radicals, stimulation of antioxidant enzymes, and chelation of transition metals [25]. As a result, melatonin assuages oxidative stress-related pathologies, reduces cellular apoptosis, and preserves the cell function. In the present study, melatonin reversed the LPS-induced reduction in dityrosine levels and suppressed nitrite levels. Dityrosine is a fluorescent molecule generated during normal post-translational processes. Because tyrosine dimerization, as well as nitration, can be affected by peroxynitrite, dityrosine is considered a biomarker of oxidatively modified proteins [53]. At peroxynitrite levels of 5 μM or less, tyrosine is almost exclusively dimerized to give dityrosine, whereas the reaction progressively shifts toward nitration at higher peroxynitrite concentrations [54]. Nitration of tyrosine residues on proteins is associated with peroxynitrite-mediated tissue injury under inflammatory conditions [55]. Our results indicate that melatonin reduced oxidative protein damage caused by LPS stimulation. NO is produced from L-arginine by the enzyme NO synthase. It is an important intracellular and extracellular signaling molecule involved in diverse biological processes, including regulation of vascular tone, neurotransmission, and the immune response, and inhibition of platelet aggregation [56]. NO is also an important cytotoxic mediator under pathological conditions. It reacts with oxygen, superoxide anions, and reducing agents to generate products with many toxic effects, causing nitrosative stress [57]. Reductions in NO levels are beneficial to cells, precluding LPS action and reducing inflammation. NO released by cultured cells into the medium is readily oxidized to nitrite [58]. Nitrite levels are significantly increased under inflammatory conditions [58]. The results of the present study reveal that levels of nitrite in the culture medium of LPS-stimulated bMECs were markedly suppressed by pretreatment with melatonin. Therefore, melatonin prevented nitrosative stress caused by LPS stimulation. We also found that melatonin activated the Nrf2 antioxidant defense pathway. Nrf2 is an important sensor of oxidants. Upon activation, Nrf2 translocates to the nucleus and interacts with the antioxidant response element to initiate transcription of HO-1 [59]. HO-1 enhances cellular resistance to oxidative stress and protects against inflammation [60]. In the present study, we found that melatonin protected bMECs from LPS-induced inflammatory and oxidant stress damage. In future studies, we will administer melatonin to dairy cows with clinical and subclinical mastitis to determine whether it has a therapeutic effect in the field.

## Conclusions

We found that melatonin treatment of bMECs attenuated the LPS-stimulated increase in pro-inflammatory cytokine, chemokine, and positive APP mRNA but increased the expression of an anti-inflammatory cytokine and a negative APP by inhibiting the TLR4 signaling pathway. In addition, melatonin inhibited oxidative stress by activating the Nrf2 antioxidant defense pathway. Finally, melatonin treatment had a beneficial effect on the viability of LPS-stimulated bMECs.

## Supporting information

S1 Fig Both 100 and 1,000 ng/mL LPS increase the expression of TNF-α and SAA mRNA in bMECs. qPCR analysis of (A) TNF-α, and (B) SAA. Data are the mean ± SD of three independent experiments. *P < 0.05 vs the control group.
(TIF)
