# Supplementary figures and images for: The anti-inflammatory and antioxidant effects of melatonin on LPS-stimulated bovine mammary epithelial cells
Source: PLoS One. 2017 May 25;12(5):e0178525. doi: 10.1371/journal.pone.0178525 (PMC5444821; doi:10.1371/journal.pone.0178525)

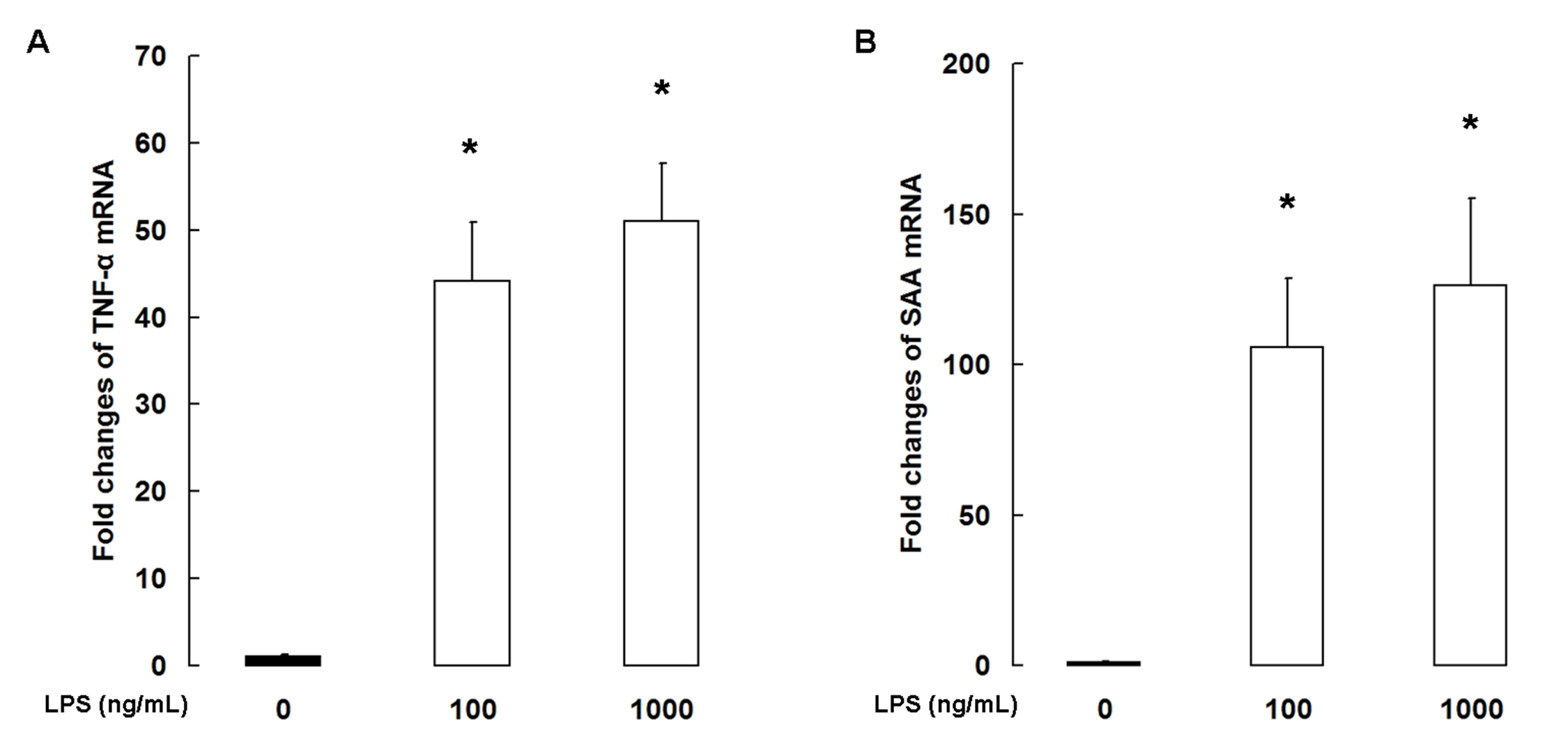

Supplement: S1 Fig — qPCR analysis of (A) TNF-α, and (B) SAA. Data are the mean ± SD of three independent experiments. *P < 0.05 vs the control group. (TIF) [file pone.0178525.s001.tif]
